# Intramuscular plasmid DNA electroporation sequesters neoantigen-specific CD8^+^ T cells in treated muscle and limits tumor infiltration

**DOI:** 10.1016/j.omton.2026.201313

**Published:** 2026-09-03

**Authors:** Andrea Bianchi, Mauro Esposito, Tiziano Giacomelli, Ilaria Esposito, Claudia Tonini, Luigi Aurisicchio, Fabio Palombo

## Main text

(Molecular Therapy: Oncology *34*, 201248; September 2026)

In the originally published article, the authors’ given names and surnames were inverted in the author list. This has now been corrected online. The authors and production apologize for this error.

