# Supplementary material for: Intramuscular plasmid DNA electroporation sequesters neoantigen-specific CD8+ T cells in treated muscle and limits tumor infiltration
Source: Mol Ther Oncol. 2026 May 28;34(3):201248. doi: 10.1016/j.omton.2026.201248 (PMC13334405; doi:10.1016/j.omton.2026.201248)
Supplement: Document S1. Figures S1–S4 [file mmc1.pdf]

OMTON, Volume *34*

Supplemental information

Intramuscular plasmid DNA electroporation sequesters neoantigen-specific CD8$^+$ T cells in treated muscle and limits tumor infiltration

Bianchi Andrea, Esposito Mauro, Giacomelli Tiziano, Esposito Ilaria, Tonini Claudia, Aurisicchio Luigi, and Palombo Fabio

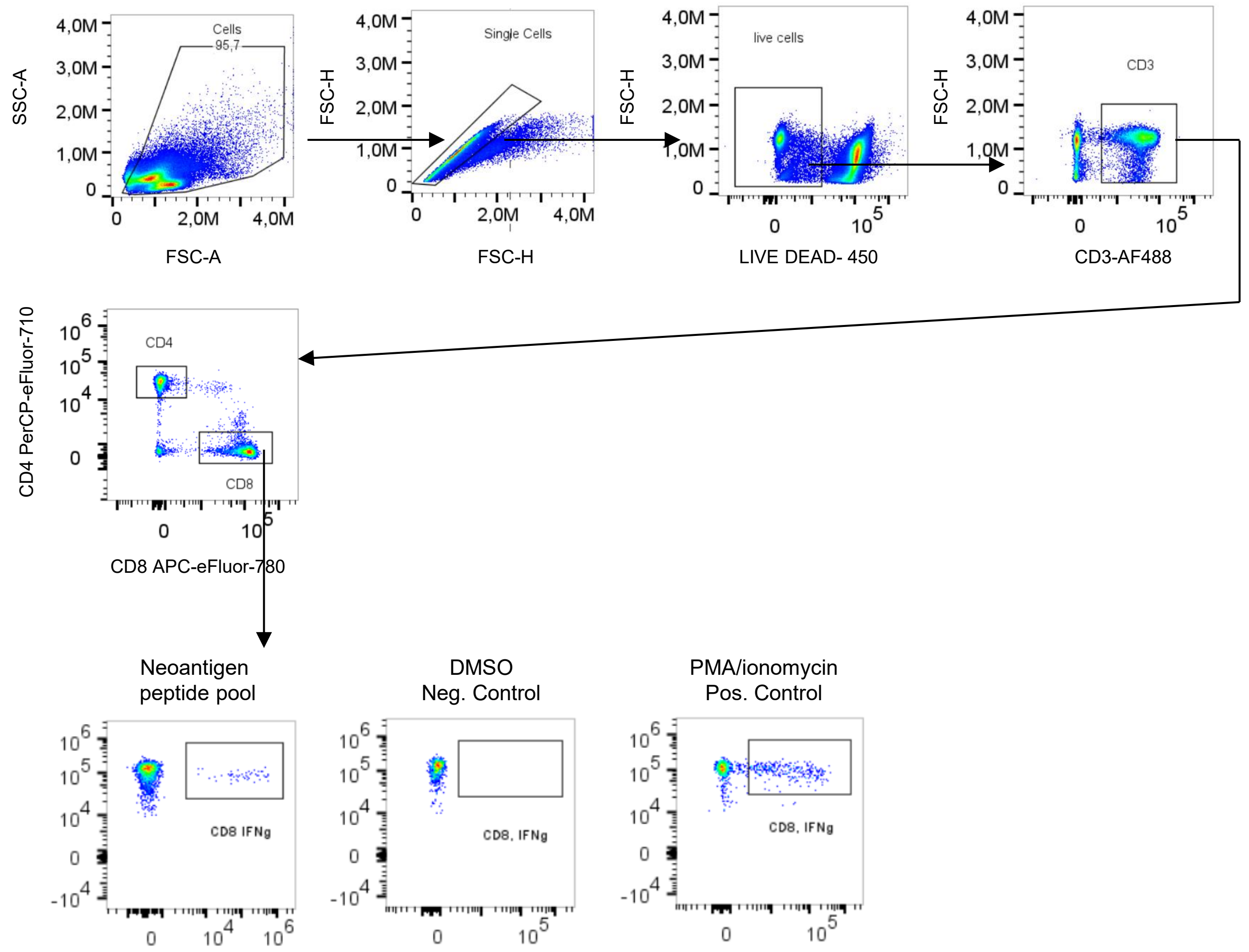

Figure S1. Representation of the gating strategy for neoantigen-specific T cells in blood sample. Isolated PBMCs were re-stimulated with the neoantigen peptide pool, DMSO (as negative control),or PMA/ionomycin (as positive control) and $CD3^{+}$ $CD8^{+}$ $IFN\gamma^{+}$ identified as consecutive gating strategy.

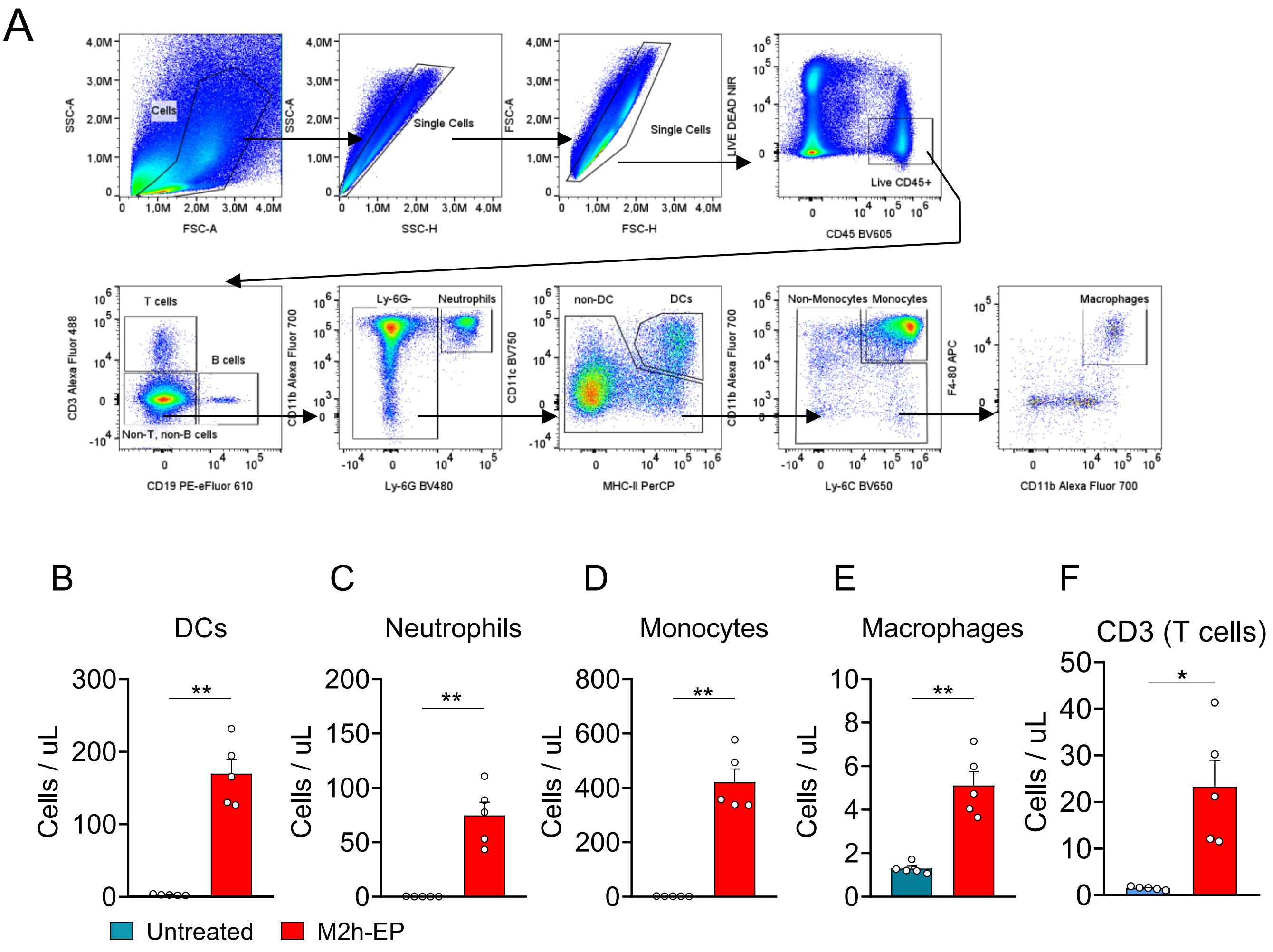

Figure S2. Representation of the gating strategy for innate and adoptive immune cells. Dendritic cells (DCs), Neutrophils, Monocytes, Macrophages, and CD3 T Cells population were anayzed in vaccinated muscles following the depicted gaing strategy 24h after vaccination (**A**) with relative quantification (**B**, **C**, **D**, **E**, **F**), n=5 (unpaired two-tailed t-test). Data are presented as mean ± SEM. *p < 0.05, **p < 0.01, ***p < 0.001, ****p < 0.0001; ns, non-significantly different.

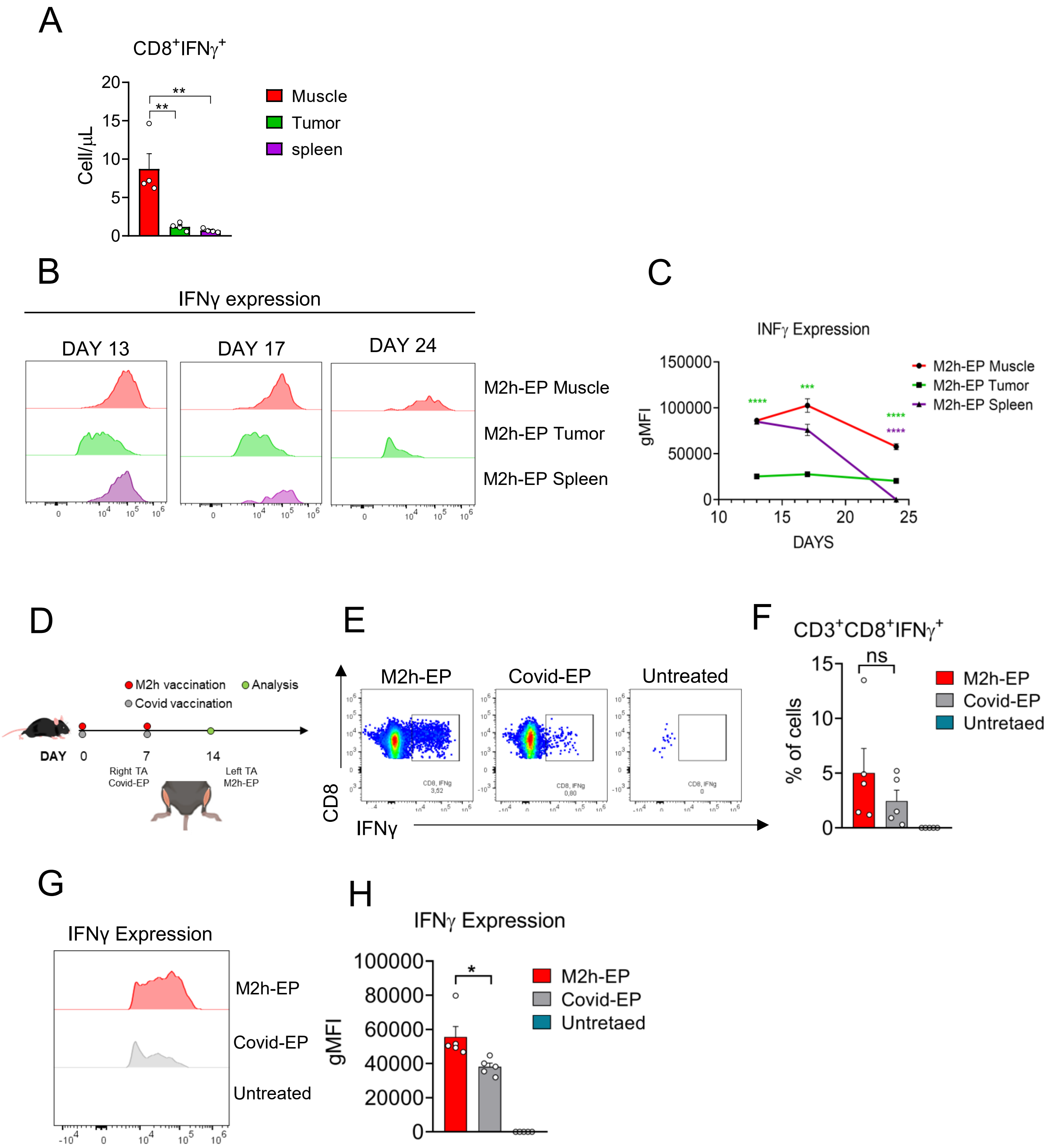

Figure S3. Quantification of neoantigen-specific T cells in different tissues. Quantification of total neoantigen-specific CD8+ T-cell (CD8+ IFNγ+) measured as cell/μL (**A**). Geometric mean fluorescence intensisty (gMFI) in CD8+ IFNγ+ T cells (**B**) and relative quantification (**C**), n= 4-5. (Ordinary one way Anova for multiple comparison). Vaccination scheme for secondary inflammation site for depot effect (**D**). Representative images of IFNγ ICS analysis (**E**) and relative quantification (**F**), n=5 (unpaired two-tailed t-test between). IFNγ expression expressed as gMFI in CD8+ CD8+ IFNγ+ T cells (**G**) and relative quantification (**H**), n= 4-5 (unpaired two-tailed t-test). All data are reported as mean ± SEM. *$p < 0.05$, **$p < 0.01$, ***$p < 0.001$, ****$p < 0.0001$ and ns= not statistically significant

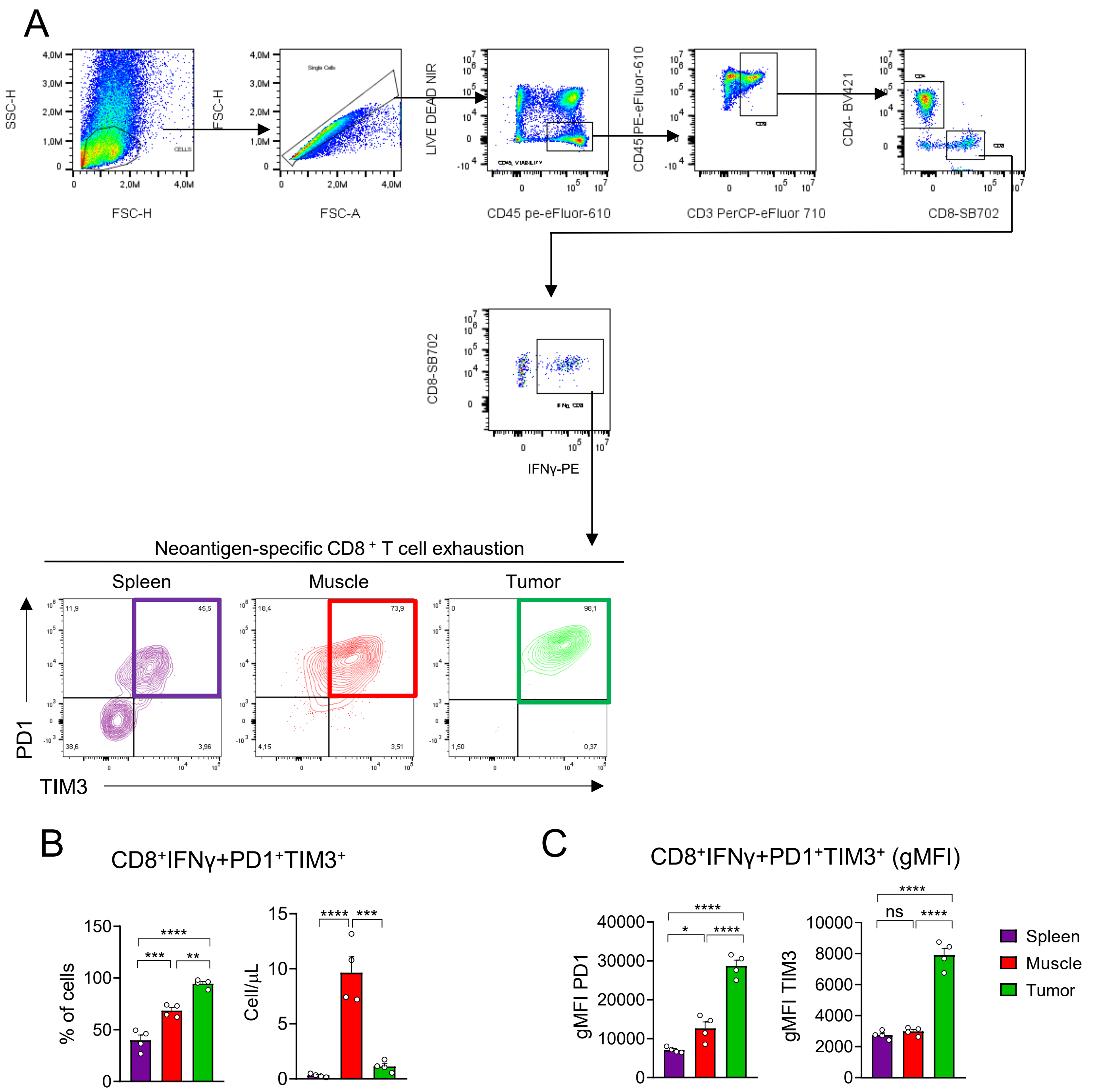

Figure S4. Flow cytometry analysis of neoantigen-specific $CD8^{+}$ T cell exhaustion. Representation of the gating strategy for the PD1 and TIM3 expression in neoantigen-specific $CD8^{+}IFN\gamma^{+}$ T cells in spleen, muscle and tumor on day 13 (**A**). Percentage and total number of $CD8^{+}IFN\gamma^{+}PD1^{+}TIM3^{+}$ (**B**). gMFI of PD1 and TIM3 in $CD8^{+}IFN\gamma+PD1^{+}TIM3^{+}$ (**C**). All data (n=4) are reported as mean ± SEM. **p* < 0.05, ***p* < 0.01, ****p* < 0.001, *****p* < 0.0001 and ns= not statistically significant (Ordinary one way Anova for multiple comparison).
