# Supplementary material for: Intramuscular plasmid DNA electroporation sequesters neoantigen-specific CD8+ T cells in treated muscle and limits tumor infiltration
Source: Mol Ther Oncol. 2026 May 28;34(3):201248. doi: 10.1016/j.omton.2026.201248 (PMC13334405; doi:10.1016/j.omton.2026.201248)
Supplement: Document S2. Article plus supplemental information [file mmc2.pdf]

Molecular Therapy
Oncology
Brief report

Intramuscular plasmid DNA electroporation sequesters neoantigen-specific CD8$^+$ T cells in treated muscle and limits tumor infiltration

Bianchi Andrea,[1] Esposito Mauro,[1] Giacomelli Tiziano,[1,2] Esposito Ilaria,[1] Tonini Claudia,[1] Aurisicchio Luigi,[1,3] and Palombo Fabio[1]

[1]Neomatrix, Rome, Italy; [2]Sapienza University, Rome, Italy; [3]Takis, Rome, Italy

DNA electroporation of neoantigen cancer vaccine is a well-established approach to induce high expression of neoantigens and likely enlarge the immune response that is shaped by the vaccination site influencing T cell trafficking. Using a DNA plasmid vaccine encoding MC38-derived neoantigens, we found that in presence of tumor, the vaccine delivery by muscle electroporation promotes extensive activation of immune-related genes and CD8$^+$ T cell enrichment in muscle together with inflammatory markers, whereas tumors show minimal transcriptional changes compared with untreated controls. Flow cytometry analysis revealed accumulation of neoantigen-specific CD8$^+$IFNγ$^+$ T cells in electroporated muscles compared to spleen or tumor, consistent with preferential localization at the vaccination site. Vaccination in a second site with an unrelated plasmid DNA electroporation partially reproduced this effect, consistent with neoantigen-independent recruitment of activated CD8$^+$ T cells. Adoptive T cells transfer further demonstrated preferential T cell homing to electroporated tissue. These findings identify electroporation-induced tissue remodeling as a major contributor associated with altered T cell localization and suggest that modulating local tissue responses may improve the systemic efficacy of DNA-based cancer vaccines.

INTRODUCTION

The neoantigen cancer vaccine (NCV) approach for precision and personalized immunotherapy is currently at clinical stage with a variety of different delivery platforms spanning from synthetic peptides[1] to genetic vaccines including mRNA formulated in lipid nanoparticles[2] and plasmid DNA (pDNA) delivered by electroporation (EP).[3] Virus-based and lipid nanoparticles-based NCV share an intrinsic immunogenicity and inflammatory properties that support the immune response against neoantigens, suggesting the inflammatory component as a crucial player for the NCV efficacy. Within this landscape, the quality and durability of immune responses against cancer neoantigens are two features strongly pursued and optimized. Within the context of pDNA vaccination, intramuscular delivery followed by EP (pDNA-EP) has emerged as one of the most effective approaches to enhance antigen expression and immunogenicity. pDNA allows flexible engineering of neoantigens, while EP facilitates DNA uptake and triggers a local inflammatory reaction that mobilizes immune cells.[4]

Given that skeletal muscle represents nearly 40% of body mass,[5] it has become a preferred target for DNA vaccination site, and the requirement for EP has been clearly established in early clinical studies.[6] However, the side effects of muscle EP on immune cell trafficking remain underexplored, despite evidences that electrical fields can modify tissue homeostasis,[7] induce transient damage,[8] and recruit immune infiltrates like those observed during muscle injury and regeneration.[9] Aligned with these observations, several papers support the idea that pDNA-EP induces an inflammatory reaction. Previous immune histochemistry analysis showed mononuclear cell infiltration in pDNA-EP-treated muscle[10] and different lymphocyte populations were characterized by flow cytometry.[11] Moreover, histological analyses revealed the recruitment of CD8$^+$ T cells to electroporated muscle.[12] More recently, RNA sequencing (RNA-seq) studies confirmed acute inflammatory and chemokine responses following pDNA-EP.[13]

Together, these observations suggest that muscle EP provokes a dynamic response that combines DNA expression with local inflammation, conditions that may favor antigen-specific T cell retention at the vaccination site rather than trafficking to distal tissues, including tumors. The persistence of antigen and inflammation at the vaccination site is recognized as a key factor shaping the outcome of cancer immunization strategies. This phenomenon, commonly referred to as the “depot effect”, describes how a local reservoir of antigen and immune activation can attract and retain T cells at the site of vaccination, sometimes at the expense of their migration to tumors.[14,15] Here, we hypothesized that pDNA-EP vaccination generates a dominant inflammatory tissue niche that is more permissive to CD8$^+$ T cell accumulation than the tumor microenvironment, including neoantigen-specific populations. Moreover, adoptive T cell transfer

Received 18 February 2026; accepted 26 May 2026;
https://doi.org/10.1016/j.omton.2026.201248.

Correspondence: Palombo Fabio, Neomatrix, Rome, Italy.
E-mail: fabio.palombo@neomatrixbiotech.com

© 2026 The Author(s).
Published by Elsevier Inc. on behalf of The American Society of Gene and Cell Therapy.
This is an open access article under the CC BY license (http://creativecommons.org/licenses/by/4.0/).

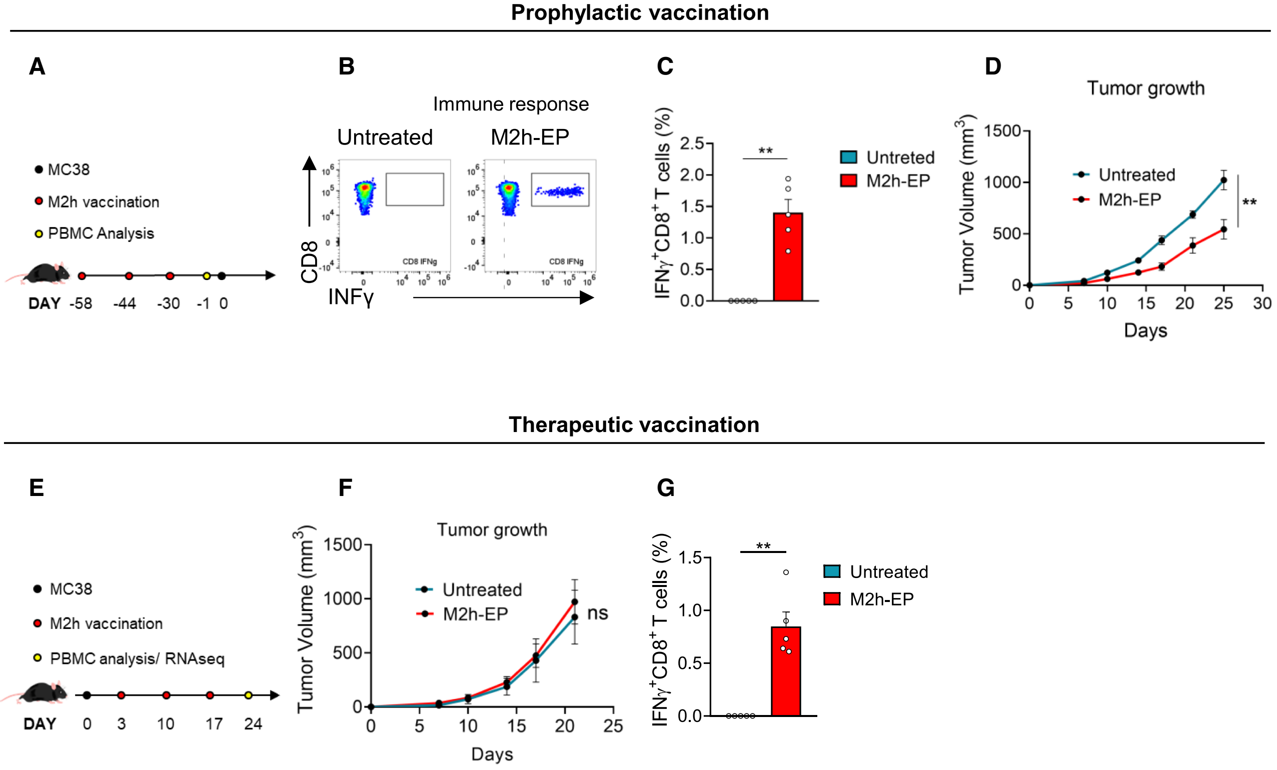

Figure 1. M2h-EP therapeutic vaccination induces systemic neoantigen-specific immune responses but fails to control tumor growth

(A) Prophylactic vaccination schedule. (B) Representative flow cytometry plots of neoantigen-specific $CD3^+CD8^+IFN\gamma^+$ T cells in peripheral blood mononuclear cells (PBMCs) measured by intracellular cytokine staining (ICS). (C) Quantification of neoantigen-specific $CD3^+CD8^+IFN\gamma^+$ T cells shown in (B). (D) Tumor growth under prophylactic conditions ($n = 5$; unpaired two-tailed *t* test with Welch's correction). (E) Therapeutic vaccination schedule. (F) Tumor growth under therapeutic conditions ($n = 5$; unpaired two-tailed *t* test with Welch's correction). (G) Quantification of neoantigen-specific $CD3^+CD8^+IFN\gamma^+$ T cells on day 24 after tumor challenge. Data are presented as mean ± SEM. $^*p < 0.05$, $^{**}p < 0.01$.

in immunodeficient mice corroborates the preferential homing of $CD8^+$ T cells in electroporated site respect tumor and spleen, supporting the evidence that inflammation, evoked by muscle EP is associated with reduced tumor infiltration of neoantigen specific $CD8^+$ T cells in a therapeutic MC38 model.

RESULTS

M2h-EP therapeutic vaccination induces systemic neoantigen-specific immune response but fails to control tumor growth in a therapeutic setting

We compared the impact of pDNA-EP vaccination on tumor growth in prophylactic vs. therapeutic setting. To this end, we used the M2h plasmid encoding MC38-derived neoantigens and helper CD4 epitopes.[16,17] In the prophylactic setting (Figure 1A), analysis of neoantigen-specific immune responses, on the day before tumor challenge, showed a significant induction of $CD3^+CD8^+IFN\gamma^+$ T cells measured by intracellular cytokine staining (ICS) flow cytometry in peripheral blood mononuclear cells (PBMCs) (Figures 1B, 1C, and S1A). According to this observation, the preventive M2h-EP vaccination, significantly reduced the tumor growth (Figure 1D). In contrast, in therapeutic setting (Figure 1E), when the vaccination started after MC38 implant, M2h-EP failed to control tumor growth (Figure 1F), despite the fact that peripheral $CD3^+CD8^+IFN\gamma^+$ neoantigen-specific immune responses were still present on day 24 (Figure 1G). These results indicate that the induction of systemic neoantigen-specific $CD8^+$ T cell responses is not sufficient to mediate tumor control in the therapeutic context.

M2h-EP therapeutic vaccination drives strong transcriptional remodeling in muscle but not in tumor

To shed light on the discrepancy, we characterized the injected site and tumor at different time points. Previous evidence has shown both natural and adoptive immune cell infiltration associated with pDNA-EP[12,18]; therefore, we analyzed muscle infiltration by flow cytometry. In our experimental condition, M2h-EP vaccination triggered local inflammation as indicated by the presence of different immune cell populations 24 h post-vaccination scarcely present in undamaged muscle. These populations included innate immune cells (dendritic cells, neutrophils, monocytes, and macrophages) as well as adaptive immune cells (T cells) (Figures S2B–S2F).

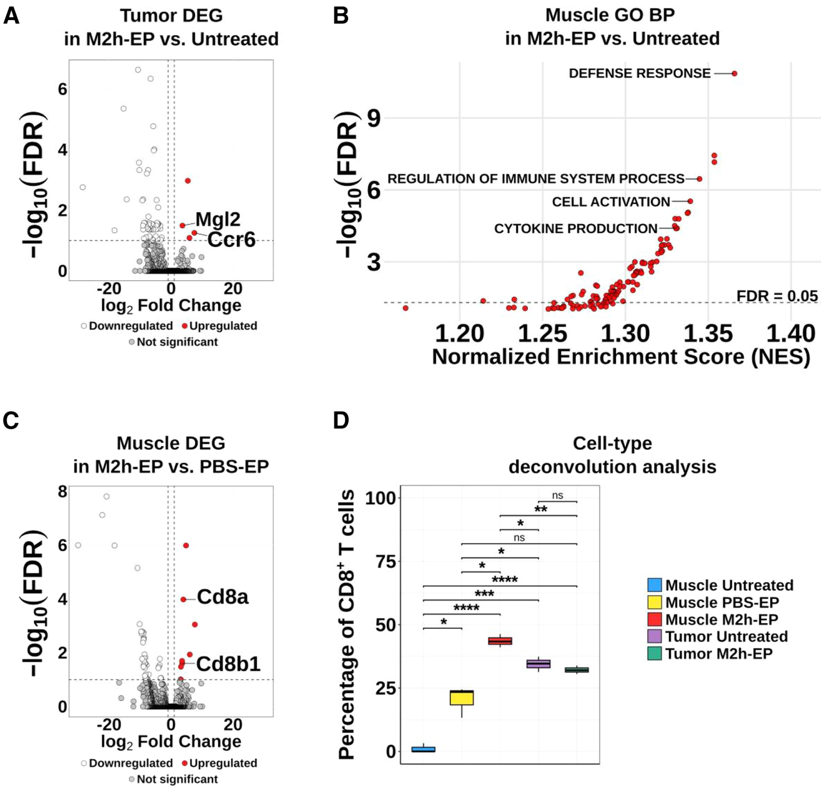

Figure 2. M2h-EP vaccination drives strong transcriptional remodeling in muscle but not in tumor

Tissues were collected on 7 days after the last vaccination corresponding to day 24 in Figure 1E (A) Volcano plot showing differentially expressed genes (DEGs) in tumors from M2h-EP–vaccinated versus untreated mice (n = 3). (B) Scatterplot of enriched Gene Ontology Biological Process (GOBP) pathways in M2h-EP vaccinated versus untreated muscle, showing normalized enrichment score (NES) versus significance ($-\log_{10}$ FDR) (n = 3). (C) Volcano plot showing DEGs in M2h-EP muscles when compared to PBS-EP treated mice (n = 3). (D) Boxplots showing inferred proportions of $CD8^+$ T cells across experimental groups (n = 3; unpaired two-tailed t test). Data are presented as mean ± SEM. $^*p < 0.05$, $^{**}p < 0.01$, $^{***}p < 0.001$, $^{****}p < 0.0001$; ns, not significant.

To characterize together the tumor and muscle microenvironments in a more comprehensive manner we compared tumors and site of vaccinations by RNA-seq at later time points under therapeutic settings (as reported in Figure 1E). On day 7 after the last vaccination, tumors isolated from vaccinated mice showed minimal transcriptional remodeling compared with untreated controls, with only a small set of differentially expressed genes (DEGs) (such as *Ccr6* and *Mgl2*) overexpressed in the tumor of vaccinated mice, and no coherent immune activation by GSEA (Figure 2A; Supplementary Table S1). In sharp contrast, M2h-EP-treated muscles displayed a broad inflammatory and immune-activation program when compared with untreated control, including enrichment of defense response, immune cell activation, and cytokine production pathways (Figure 2B; Supplementary Table S2). As control we looked at muscle treated with PBS-EP. A less organized signature was observed when compared to untreated controls, consistent with pDNA-EP driven tissue stress responses previously reported.[12] Direct comparison between M2h-EP and PBS-EP muscles revealed increased expression of T-cell-associated transcripts (Cd8a/Cd8b1) and enrichment of immune response pathways in M2h-EP treated muscles (Figure 2C; Supplementary Table S3). Deconvolution supported a marked increase in $CD8^+$ T cell representation in M2h-EP muscle, exceeding that inferred in tumors (Figure 2D; Supplementary Table S4). Together, these data suggest that M2h-EP creates a muscle inflammatory niche permissive for $CD8^+$ T cell accumulation, while tumors remain comparatively unchanged.

Neoantigen-specific $CD3^+CD8^+$ T cells preferentially accumulate and persist at the EP site

To corroborate the RNA-seq data and evaluate whether neoantigen-specific $CD8^+$ T cells are retained in muscle, we quantified $CD3^+CD8^+$ T cell infiltration and function by flow cytometry in muscle, tumor, and spleen on days 3, 7, and 14 after the last vaccination (corresponding to day 13, 17, and 24 after tumor implant) (Figure 3A). Total $CD3^+CD8^+$ T cells were significantly enriched in electroporated muscles relative to tumors across most of the time points analyzed, with the strongest differences during early inflammation and remodeling stage (days 3–7 after last vaccination; Figure 3B).

Notably, after neoantigen-peptide-pool *ex vivo* stimulation, the percentage of neoantigen-specific $CD3^+CD8^+IFN\gamma^+$ T cells and total number (Figure S3A) were consistently more abundant in muscle than in spleen or in tumor (Figures 3C and 3D). The geometric mean fluorescence intensity (gMFI) of IFNγ was also higher in muscle, whereas responses in tumor remained constant over time (Figures S3B and S3C). We then asked whether these cells were exhausted. Our high-dimensional analysis revealed a clear activation gradient. While splenic T cells presenting a resting state ($PD1^-TIM3^-$), the vaccinated muscle acts as a massive primary hub for effector generation. Although 70% of these cells expressed activation/exhaustion markers (Figures S4A and S4B), the absolute number of $CD3^+CD8^+IFN\gamma^+$ T cells was 7-fold higher than in the tumor (Figure S3A), and they maintained a robust IFNγ output. This indicated that the muscle provides a superior reservoir of functional effectors compared to the tumor microenvironment, where the $PD1^+TIM3^+$ (98%) eventually correlated with terminal dysfunction. In line with these results, the gMFI values of exhaustion markers increased from the spleens to the tumors (Figure S4C). These findings support a functional cellular "depot effect" in which M2h-EP

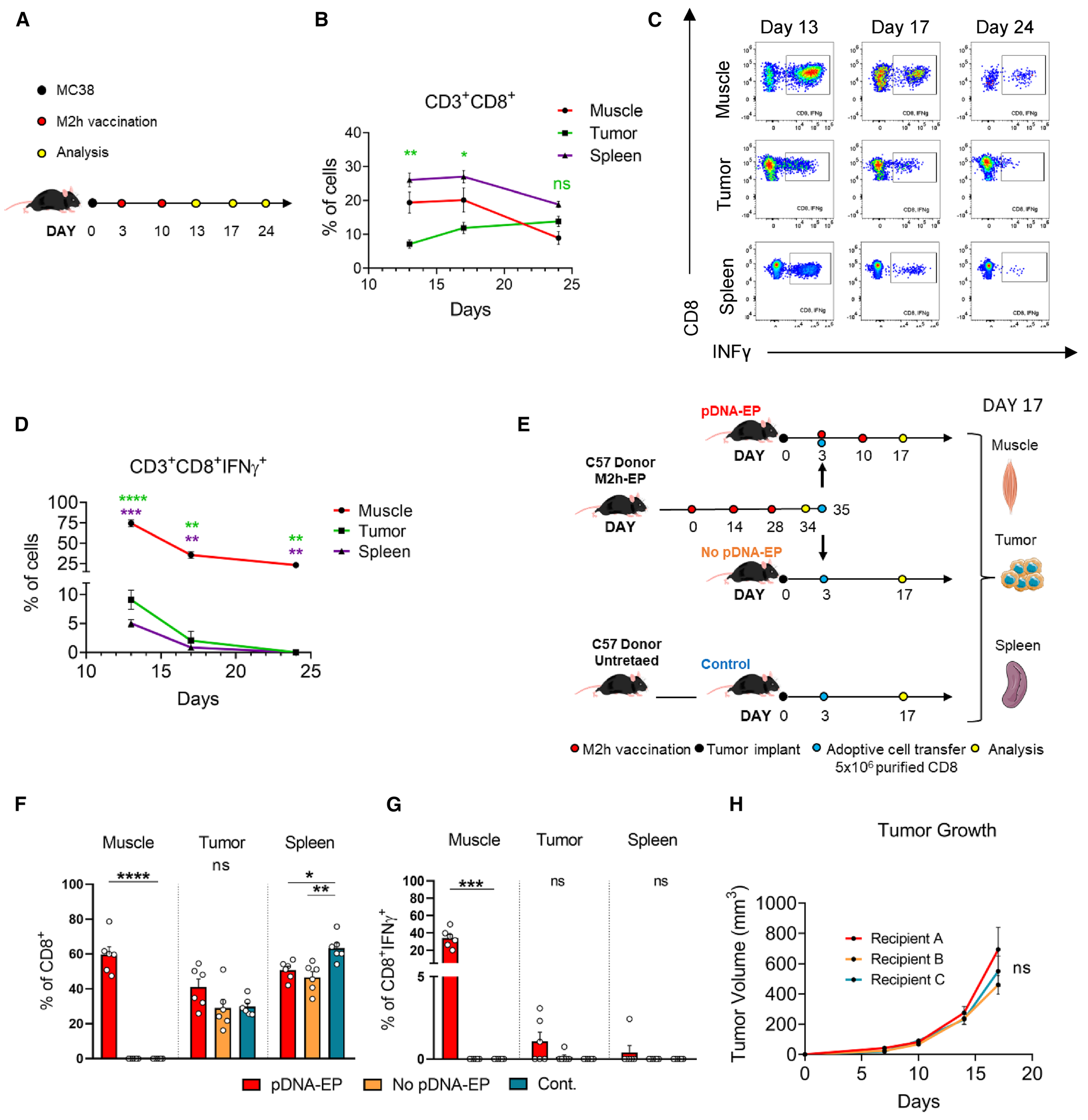

Figure 3. Neoantigen-specific CD3$^+$CD8$^+$ T cells preferentially accumulate and persist at the electroporation site

(A) Therapeutic vaccination scheme. (B) Flow cytometry quantification of total CD3$^+$CD8$^+$ T cells in muscle, tumor, and spleen at indicated time points after vaccination (n = 4–5; ordinary one-way ANOVA with multiple comparisons). (C) Representative flow cytometry plots of neoantigen-specific CD3$^+$CD8$^+$IFNγ^+ T cells measured by intracellular cytokine staining (ICS) in muscle, tumor, and spleen. (D) Quantification of neoantigen-specific CD3$^+$CD8$^+$IFNγ^+ T cells shown in (C) (n = 4–5; ordinary one-way ANOVA with multiple comparisons). (E) Schematic representation of the adoptive T cell transfer experiment. (F) Flow cytometry quantification of total CD3$^+$CD8$^+$ T cells in muscle, tumor, and spleen following adoptive transfer (n = 6; ordinary one-way ANOVA with multiple comparisons). (G) Quantification of neoantigen-specific CD3$^+$CD8$^+$IFNγ^+ T cells following adoptive transfer (n = 6; ordinary one-way ANOVA with multiple comparisons). (H) Tumor growth following adoptive T cell transfer (n = 6; ordinary one-way ANOVA with multiple comparisons). Data are presented as mean ± SEM. *p < 0.05, **p < 0.01, ***p < 0.001, ****p < 0.0001; ns, not significant.

is associated with preferential localization and persistence of vaccine-elicited effector CD8$^+$ T cells at the vaccination site. The impact of vaccine target expression on the homing of induced T cell responses was addressed by EP with an unrelated plasmid (COVID-eVax,[19] hereafter Covid, not containing MC38 neoantigens) in a second muscle (Figure S3D). Surprisingly, the neoantigen-specific CD3$^+$CD8$^+$IFNγ$^+$ T cells accumulated also in the second inflammation site (Figures S3E and S3F) albeit at reduced magnitude compared with the M2h-EP vaccination site, and expressed lower level of IFNγ (Figures S3G and S3H). This evidence supports a model in which inflammation and tissue remodeling driven by pDNA-EP can recruit activated, antigen-experienced CD8$^+$ T cells from the periphery, independent of local antigen production, consistent with pDNA-EP acting as a dominant tissue "sink" for circulating effectors.

Adoptive T cell transfer reveals preferential homing of neoantigen-specific CD8$^+$ T cells to electroporated muscle

Since the kinetic of induction of neoantigen-specific immune responses are different in prophylactic and therapeutic settings, we adopted a T cell transfer experiment from vaccinated or unvaccinated mice to Rag2$^-$/$^-$ tumor-bearing recipients. In this context, we asked whether pDNA-EP sites actively attract and maintain neoantigen-specific T cells. Equal number of CD8-enriched T cells from vaccinated donors (C57BL/6 mice) were transferred into immunodeficient Rag2$^-$/$^-$ mice, with or without a pDNA-EP-treated muscle site. As negative control, CD8 enriched cells from unvaccinated C57BL/6 donor mice were transferred into Rag2$^-$/$^-$ tumor-bearing recipients without EP site (Figure 3E).

On day, 17 mice were sacrificed to analyze tumor, spleen, and muscles for the presence of CD3$^+$CD8$^+$ T cells and neoantigens-specific CD3$^+$CD8$^+$IFNγ$^+$ T cells. As expected, CD3$^+$CD8$^+$ T cells were consistently detected at variable frequency in the spleen and tumor, while in muscle they were detected at high frequency only if treated with pDNA-EP (Figure 3F). Strikingly, the neoantigen specific CD3$^+$CD8$^+$IFNγ$^+$ T cells were preferentially detected in recipients receiving pDNA-EP and accumulated at high frequency in muscle, with minimal to no detectable cells in tumor and spleen (Figure 3G). Lack of neoantigen specific CD3$^+$CD8$^+$IFNγ$^+$ T cells in the absence of pDNA-EP may suggest a requirement for persistence/survival of highly activated effectors in this system as previously suggested.[14,20] Tumor growth effects were modest in this transfer setting (Figure 3H), likely reflecting limited effector numbers and the absence of checkpoint blockade inhibitors.[21]

DISCUSSION

We investigated muscle immune infiltration in relation to tumor growth, starting from the observation that prophylactic—but not therapeutic—pDNA-EP vaccination significantly delayed tumor progression despite the induction of comparable neoantigen-specific immune responses (Figure 1). One possible explanation is that, in the presence of established tumors, pDNA-EP alters CD8$^+$ T cell trafficking and localization, thereby limiting effective tumor infiltration. To explore this hypothesis, we characterized both muscle and tumor tissues by RNA-seq (Figure 2), confirming by flow cytometry that our pDNA-EP conditions induced immune infiltration comparable to that reported previously in the muscle (Figure S2). Notably, at later time points, the most significantly deregulated genes in muscle were associated with CD8$^+$ T cell signatures. Guided by these transcriptomic findings, we further analyzed adoptively transferred T cells by flow cytometry, with a specific focus on neoantigen-specific populations.

The marked accumulation of neoantigen-specific CD8$^+$ T cells in electroporated muscle, but not in tumors, provides a plausible explanation for the lack of antitumor efficacy in the therapeutic setting (Figure 3). EP of a second muscle with an unrelated plasmid resulted in significant but reduced frequency of infiltrating neoantigen-specific CD3$^+$CD8$^+$IFNγ$^+$ T cells (Figure S3), suggesting that local neoantigen expression contributes, at least in part, to this retention. These data suggest that one mechanism contributing to the inefficient antitumor activity of pDNA-EP vaccination is the preferential retention of neoantigen-specific CD3$^+$CD8$^+$IFNγ$^+$ T cells at the EP site. This phenomenon, commonly referred to as a "depot effect," is consistent with previous reports describing prolonged T cell sequestration at vaccination sites following peptide vaccination formulated in incomplete Freund's adjuvant[15] or after mRNA-LNP vaccination.[14]

Importantly, adoptive T cell transfer experiments further confirmed preferential homing and persistence of neoantigen-specific T cells within pDNA-EP-treated muscle, with minimal infiltration into tumors and only in the presence of EP (Figure 3). These results not only account for differences in immune-response kinetics between prophylactic and therapeutic settings but also reveal a previously unrecognized effect of EP associated with enhanced persistence of neoantigen-specific T cells. Interestingly, following adoptive T cell transfer in the absence of EP, neoantigen-specific CD8$^+$ T cells were no longer detectable on day 14, yet a marginal antitumor effect was still observed. This finding suggests that maintaining effective levels of pDNA-EP-induced effector T cells may require additional immune modulation. Consistent with this interpretation, previous studies targeting MC38 neoantigens reported a significant antitumor activity only when T cell transfer was combined with immune checkpoint blockade, such as anti-PD-L1 treatment.[21]

Muscle EP induces transient tissue damage that elicits inflammatory signals governing muscle repair, a highly coordinated process involving extensive crosstalk between muscle stem cells and immune cells and typically requiring 2–3 weeks to resolve.[22] Repeated pDNA-EP administration during this healing phase may generate an excessive inflammatory burden with systemic consequences, potentially including impaired antitumor immunity. To mitigate unintended inflammation, alternative strategies could be implemented, such as modifying EP parameters, spacing or rotating injection sites, or combining pDNA-EP with interventions that promote tumor homing or overcome intramuscular T cell retention. From a translational perspective, this duality presents both opportunities and challenges. While muscle EP enables efficient *in situ* transfection and serves as a

temporary antigen-production hub, local inflammation may inadvertently sequester effector T cells, limiting their tumor-directed migration. Notably, antitumor efficacy was observed on later time points, when inflammation at the EP site is likely resolved, further supporting the importance of temporally separating pDNA-EP-induced inflammation from tumor growth.

Finally, we cannot exclude that the depot effect described here may be more pronounced in subcutaneous tumor models, whereas in more vascularized settings—such as lung metastases—it may be attenuated. Additional studies in alternative tumor models and larger animals cohort will be required to determine the generalizability and clinical relevance of this phenomenon.

MATERIALS AND METHODS

Mice and ethics

Female C57BL/6 and Rag2$^{-/-}$ mice (6–7 weeks old) were housed under standard conditions. All procedures were approved by the Italian Ministry of Health ethical committee (authorization A69A0.150).

Cell culture

MC38 colon carcinoma cells (Kerafast, ENH204-FP) were maintained in DMEM (Invitrogen cat. 41966-029) supplemented with 10% Fetal Bovine Serum (Invitrogen cat. A5256701) and 1% Pen/strep (Invitrogen cat. 15070-063) at 37°C with 5% CO_2.

pDNA-EP and tumor challenge

M2h,[17] a vaccine expressing MC38 specific neoantigens (Adpgk, Dpagt1, Reps1, Tmem135, Spire1, Wbp7, Hace1, and Nle1) and the helper CD4 epitopes (FNNFTVSFWLRVPKVSASHLE, AKFVAAWTLKAAAW, andAWLEAQEEEEVGF) and COVID-eVax[19] plasmids were intramuscularly injected in *Tibialis Anterior* (10 μg per mouse) and electroporated using Igea Cliniporator system with the following parameters: Low voltage 110 V, number of pulses 8, length 20 milliseconds, pause 120 milliseconds. MC38 cells (5×10^5) were implanted subcutaneously in the flank and tumors were measured by caliper twice a week. Tumor volume was calculated using the formula $V = L \times W \times W \times 0.5$.

Tissue harvest

Muscles were minced and digested into 2 mL of digestion buffer containing 2.4U of Dispase II (Roche, cat. 04942078001), 2 μg/μL of Collagenase A (Roche, cat. 10103586001), 10 μg/mL DNaseI Roche (cat. 10104159001, 5 mM $MgCl_2$ + 0.4 mM of $CaCl_2$) in PBS, and incubated for 30 min at 37°C mixing the tubes every 10 min. Cell suspension was filtered through 70 μm strainers, washed in complete medium and filtered again through 30 μm strainers. Cells were plated into 96 well in a final volume of 100 μL of RPMI+ and incubated 37°C with 5% CO_2 before fresh surface staining or *ex vivo* restimulation.

Tumor processing

Tumors were minced and digested by using the Tumor Dissociation Kit (Miltenyi cat. 130.096.730) for 40 min at 37°C, mixing the tubes every 10 min, filtered through 70 μm strainers, and washed with 10 mL of RPMI. Cells were lysed with 1 mL of ACK, followed by 2 washes with RPMI+.

Spleen processing

Splenocytes were prepared as previously shown.[23] Briefly, spleens were mechanically dissociated through 70 μm strainers, washed twice with 10 mL RPMI+, incubated with 1 mL ACK lysis buffer (Gibco, cat. 10492) for 5 min at room temperature, diluted in RPMI, and washed again in complete medium.

Blood processing

Blood was collected by submandibular bleeding and incubated with 4 mL ACK lysis buffer for 10 min at room temperature, centrifuged, and subjected to two additional erythrocyte lysis steps. Cell pellets were resuspended in 100 μL RPMI+ for subsequent flow cytometry analysis.

Flow cytometry

Surface staining. Cells were incubated with the following antibodies: anti-Fcγ receptor (BD, cat. 553142); NIR for viability (Invitrogen, cat. L349756) (1:8000); CD45-BV605 (BD, cat. 563053) (1:200), CD3-AF488 (Invitrogen cat.53-0031-82) (1:100); CD19-PE-eFluor610 (Invitrogen, cat.61-0193-82) (1:80); CD11b-Alexa-Fluor700 (Invitrogen, cat.56-0112-82) (1:100); CD11c-BV750 (BioLegend, cat.117357) (1:150); Ly6C-BV650 (BD Biosciences, cat.755194) (1:200); Ly6G-BV480 (Invitrogen, cat. 41-496-6882) (1:200); MHC-II-PerCP (BioLegend, cat. 107623) (1:300); F4-80-APC (Invitrogen, 17-4801-80) (1:50). For analysis of IFN-γ-producing cells, we followed a previously described protocol.[23] Briefly, 1×10^6 cells from spleens, tumors, and muscles were stimulated overnight with the neoantigen peptide pool at the final concentration of 5 μg/mL. DMSO and PMA/ionomycin were used as negative and positive controls, respectively. After the over-night stimulation in the presence of protein transport inhibitor (Invitrogen, cat. 00-4980-03), the cells were incubated with the following reagents: anti-Fcγ receptor (BD, cat. 553142) (1:100); Fixable viability dye −450 (BD, cat. 562247) (1:4,000) or NIR (Invitrogen, cat. L349756) (1:8000), CD45-BV605 (BD, cat. 563053) (1:200) or PE-eFluor610 (Invitrogen, cat. 61-0451-82) (1:600), CD3-AF488 (Invitrogen cat.53-0031-82) (1:100) or PerCP-eFluor-710 (Invitrogen, cat. 46-0032-80) (1:160), CD4- PerCP-eFluor710 (Invitrogen cat.46-0042-82) (1:200) or BV570 (BioLegend, cat. 100541) (1:200), CD8-APC-eFluor780 (Invitrogen cat.470081-82) (1:200) or SB702 (Invitrogen, cat. 67-0081-82) (1:200), CD279(PD-1)-APC Fire810 (BioLegend, cat. 135251) (1:80), CD366(TIM3)-PE-Cy7 (Invitrogen, cat. 25-5870-82), BD Cytofix/cytoperm (BD, cat.51-2090KZ); BD perm wash 10× (BD cat.51-2091KZ); anti-IFNγ-PE (Invitrogen, cat.12-7311-82) (1:100).

Samples were acquired through a Northern Lights flow cytometer (Cytek), and data were analyzed using Cytek software according to the gating strategy depicted in Figures S1 and S2 and representative images were created on FlowJo software. When present, signal of

INFγ in DMSO-stimulated samples was subtracted from peptide-stimulated conditions.

Adoptive immune cell transfer

One week after the last vaccination (Figures 3E) 3 × 10^6 $CD8^+$ T cells from hindlimb draining lymph nodes (iliac, inguinal and poplitei) and 2 × 10^6 $CD8^+$ T-splenocytes were collected from C57BL/6 donor mice, and enriched with Easy sep Mouse $CD8^+$ T cell Isolation Kit (STEMCELL cat.19853 A). 5 × 10^6 enriched $CD8^+$ T cells were injected intravenously into $Rag2^{-}/^{-}$ tumor-bearing recipient mice.

RNA-seq and analysis

RNA from muscle and tumor tissues was extracted by homogenization using a TissueRuptor II (QIAGEN, cat. 9002756) in TRIzol reagent (Invitrogen, cat. 15596026), followed by RNA isolation according to the manufacturer's guidelines.

RNA-seq data were generated from two independent sequencing runs performed by Genomix4Life using a paired-end sequencing strategy with a read length of 2 × 100 bp and Novogene using a paired-end read length of 2 × 150 bp. In both cases, libraries were prepared using standard poly(A)+ mRNA enrichment protocols according to the respective manufacturer's guidelines and sequenced on Illumina platforms. RNA extraction, library preparation, alignment, differential expression analysis, and gene set enrichment analysis were performed using established pipelines (STAR/DESeq2/fgsea).[24–27] Batch correction for the two sequencing runs was performed as previously described.[28] Immune cell deconvolution was conducted using the Seq-ImmuCC mouse reference method.[29]

Raw read quality was assessed using FastQC.[30] The mm39 mouse reference genome and mm39.ncbiRefSeq.gtf annotation were obtained from the UCSC Genome Browser.[31] Two STAR indexes were generated using –sjdbOverhang 99 or 149 (to match the respective paired-end read lengths), and reads were aligned using STAR[24] with –twopassMode Basic and –quantMode GeneCounts.

Differentially expressed genes were detected through DESeq2[25] R package and defined as those with adjusted p value (FDR) < 0.1 and $|\log_2$ fold change$| > 1$. Batch effects were corrected using ComBat-seq[26] (sva R package).[27] Functional enrichment analysis was performed with fgsea,[28] ranking genes by the DESeq2 "stat" value, focusing on GO Biological Process gene sets (adjusted $p \leq 0.1$). Immune cell proportions were inferred after TPM normalization using Seq-ImmuCC[29] (LLSR model) and compared using pairwise t tests with FDR correction.

Statistics data are reported as mean ± SEM (specified in Figure legends). Statistical analysis was performed by using one-way ANOVA for multiple comparison or Student's t test. Significance thresholds were defined as $^*p < 0.05$, $^{**}p < 0.01$, $^{***}p < 0.001$, $^{****}p < 0.0001$ and ns = not significant.

DATA AND CODE AVAILABILITY

RNA-seq datasets (supplementary Tables S1–S4) are available in Zenodo: https://doi.org/10.5281/zenodo.18432558.

ACKNOWLEDGMENTS

We thank the staff of Plaisant for animal facility support and Takis SRL for providing M2h plasmids. This work was supported by Lazio Innova CUP F89J23001120007 (P.F.).

DECLARATION OF GENERATIVE AI AND AI-ASSISTED TECHNOLOGIES IN THE WRITING PROCESS

During the preparation of this work the author(s) used ChatGPT to keep with clarity and consistency. After using this tool/service, the author(s) reviewed and edited the content as needed and take(s) full responsibility for the content of the published article.

AUTHOR CONTRIBUTIONS

P.F. and B.A. conceived the study. B.A., E.I., T.C., A.L., and G.T. performed experiments. E.M. analyzed data. B.A., E.M., and P.F. wrote and reviewed the manuscript.

DECLARATION OF INTERESTS

T.C., B.A., E.M., and E.I. are employees of Neomatrix. A.L. and P.F. are shareholders of Neomatrix.

SUPPLEMENTAL INFORMATION

Supplemental information can be found online at https://doi.org/10.1016/j.omton.2026.201248.

REFERENCES

1. Ott, P.A., Hu-lieskovan, S., Chmielowski, B., Demario, M., Gaynor, R.B., Srinivasan, L., Ott, P.A., Hu-lieskovan, S., Chmielowski, B., Govindan, R., et al. (2020). Article A Phase Ib Trial of Personalized Neoantigen Therapy Plus Anti-PD-1 in Patients with Advanced Melanoma , Non-small Cell Lung Cancer , or Bladder Cancer. Cell *183*, 347–362.e24. https://doi.org/10.1016/j.cell.2020.08.053.
2. Weber, J.S., Khattak, M.A., Carlino, M.S., Meniawy, T., Taylor, M.H., Ansstas, G., Kim, K.B., McKean, M., Sullivan, R.J., Faries, M.B., et al. (2024). Individualized neoantigen therapy mRNA-4157 (V940) plus pembrolizumab in resected melanoma: 3-year update from the mRNA-4157-P201 (KEYNOTE-942) trial. J. Clin. Oncol. *42*, LBA9512. https://doi.org/10.1200/JCO.2024.42.17_suppl.LBA9512.
3. Kinkead, H.L., Hopkins, A., Lutz, E., Wu, A.A., Yarchoan, M., Cruz, K., Woolman, S., Vithayathil, T., Glickman, L.H., Ndubaku, C.O., et al. (2018). Combining STING-based neoantigen-targeted vaccine with checkpoint modulators enhances antitumor immunity in murine pancreatic cancer. JCI Insight *3*, e122857. https://doi.org/10.1172/jci.insight.122857.
4. Young, J.L., and Dean, D.A. (2015). Electroporation-mediated gene delivery. Adv. Genet. *89*, 49–88. https://doi.org/10.1016/bs.adgen.2014.10.003.
5. Millozzi, F., Milán-Rois, P., Sett, A., Delli Carpini, G., De Bardi, M., Gisbert-Garzarán, M., Sandonà, M., Rodríguez-Díaz, C., Martínez-Mingo, M., Pardo, I., et al. (2025). Aptamer-conjugated gold nanoparticles enable oligonucleotide delivery into muscle stem cells to promote regeneration of dystrophic muscles. Nat. Commun. *16*, 577. https://doi.org/10.1038/s41467-024-55223-9.
6. Trimble, C.L., Morrow, M.P., Kraynyak, K.A., Shen, X., Dallas, M., Yan, J., Edwards, L., Parker, R.L., Denny, L., Gi, M., et al. (2015). Safety , e ffi cacy , and immunogenicity of VGX-3100 , a therapeutic synthetic DNA vaccine targeting human papillomavirus 16 and 18 E6 and E7 proteins for cervical placebo-controlled phase 2b trial. Lancet *386*, 2078–2088.
7. Blažič, A., Guinard, M., Leskovar, T., O'Connor, R.P., and Rems, L. (2025). Long-term changes in transmembrane voltage after electroporation are governed by the interplay between nonselective leak current and ion channel activation. Bioelectrochemistry *161*, 108802. https://doi.org/10.1016/j.bioelechem.2024.108802.
8. Hartikka, J., Sukhu, L., Buchner, C., Hazard, D., Bozoukova, V., Margalith, M., Nishioka, W.K., Wheeler, C.J., Manthorp, M., and Sawdey, M. (2001). Electroporation-facilitated delivery of plasmid DNA in skeletal muscle: plasmid

dependence of muscle damage and effect of poloxamer 188. Mol. Ther. *4*, 407–415. https://doi.org/10.1006/mthe.2001.0483.

9. Derenne, A., Tassin, A., Nguyen, T.H., De Roeck, E., Jenart, V., Ansseau, E., Belayew, A., Coppée, F., Declèves, A.-E., and Legrand, A. (2020). Induction of a local muscular dystrophy using electroporation in vivo: an easy tool for screening therapeutics. Sci. Rep. *10*, 11301. https://doi.org/10.1038/s41598-020-68135-7.

10. Grønevik, E., Tollefsen, S., Bjoner Sikkeland, L.I., Haug, T., Tjelle, T.E., and Mathiesen, I. (2003). DNA transfection of mononuclear cells in muscle tissue. J. Gene Med. *5*, 909–917. https://doi.org/10.1002/jgm.416.

11. Mann, C.J., Anguela, X.M., Montané, J., Obach, M., Roca, C., Ruzo, A., Otaegui, P., Mir, L.M., and Bosch, F. (2012). Molecular signature of the immune and tissue response to non-coding plasmid DNA in skeletal muscle after electrotransfer. Gene Ther. *19*, 1177–1186. https://doi.org/10.1038/gt.2011.198.

12. Chiarella, P., Massi, E., De Robertis, M., Sibilio, A., Parrella, P., Fazio, V.M., and Signori, E. (2008). Electroporation of skeletal muscle induces danger signal release and antigen-presenting cell recruitment independently of DNA vaccine administration. Expert Opin. Biol. Ther. *8*, 1645–1657. https://doi.org/10.1517/14712598.8.11.1645.

13. Sales Conniff, A., Tur, J., Kohena, K., Zhang, M., Gibbons, J., and Heller, L.C. (2022). Transcriptomic Analysis of the Acute Skeletal Muscle Effects after Intramuscular DNA Electroporation Reveals Inflammatory Signaling. Vaccines (Basel) *10*, 2037. https://doi.org/10.3390/vaccines10122037.

14. Baharom, F., Ramirez-Valdez, R.A., Tobin, K.K.S., Yamane, H., Dutertre, C.A., Khalilnezhad, A., Reynoso, G.V., Coble, V.L., Lynn, G.M., Mulè, M.P., et al. (2021). Intravenous nanoparticle vaccination generates stem-like TCF1+ neoantigen-specific CD8+ T cells. Nat. Immunol. *22*, 41–52. https://doi.org/10.1038/s41590-020-00810-3.

15. Hailemichael, Y., Woods, A., Fu, T., He, Q., Nielsen, M.C., Hasan, F., Roszik, J., Xiao, Z., Vianden, C., Khong, H., et al. (2018). Cancer vaccine formulation dictates synergy with CTLA-4 and PD-L1 checkpoint blockade therapy. J. Clin. Investig. *128*, 1338–1354. https://doi.org/10.1172/JCI93303.

16. Aurisicchio, L., Salvatori, E., Lione, L., Bandini, S., Pallocca, M., Maggio, R., Fanciulli, M., Nicola, F.D., Goeman, F., Ciliberto, G., et al. (2019). Poly-specific neoantigen-targeted cancer vaccines delay patient derived tumor growth. J. Exp. Clin. Cancer Res. *4*, 1–13. https://doi.org/10.1186/s13046-019-1084-4.

17. Salvatori, E., Lione, L., Compagnone, M., Pinto, E., Conforti, A., Ciliberto, G., Aurisicchio, L., and Palombo, F. (2022). Neoantigen cancer vaccine augments anti-CTLA-4 efficacy. NPJ Vaccines *7*, 15. https://doi.org/10.1038/s41541-022-00433-9.

18. Calvet, C.Y., André, F.M., and Mir, L.M. (2014). Dual therapeutic benefit of electroporation-mediated DNA vaccination in vivo: Enhanced gene transfer and adjuvant activity. Oncoimmunology *3*, 37–41. https://doi.org/10.4161/onci.28540.

19. Conforti, A., Marra, E., Palombo, F., Roscilli, G., Ravà, M., Fumagalli, V., Muzi, A., Maffei, M., Luberto, L., Lione, L., et al. (2022). COVID-eVax, an electroporated DNA vaccine candidate encoding the SARS-CoV-2 RBD, elicits protective responses in animal models. Mol. Ther. *30*, 311–326. https://doi.org/10.1016/j.ymthe.2021.09.011.

20. Hailemichael, Y., Dai, Z., Jaffarzad, N., Ye, Y., Medina, M.A., Huang, X.F., Dorta-Estremera, S.M., Greeley, N.R., Nitti, G., Peng, W., et al. (2013). Persistent antigen at vaccination sites induces tumor-specific CD8+ T cell sequestration, dysfunction and deletion. Nat. Med. *19*, 465–472. https://doi.org/10.1038/nm.3105.

21. Liu, L., Chen, J., Zhang, H., Ye, J., Moore, C., Lu, C., Fang, Y., Fu, Y.X., and Li, B. (2022). Concurrent delivery of immune checkpoint blockade modulates T cell dynamics to enhance neoantigen vaccine-generated antitumor immunity. Nat. Cancer *3*, 437–452. https://doi.org/10.1038/s43018-022-00352-7.

22. Bianchi, A., Mozzetta, C., Pegoli, G., Lucini, F., Valsoni, S., Rosti, V., Petrini, C., Cortesi, A., Gregoretti, F., Antonelli, L., et al. (2020). Dysfunctional polycomb transcriptional repression contributes to lamin A/C – dependent muscular dystrophy. J. Clin. Invest. *130*, 2408–2421.

23. Lione, L., Salvatori, E., Petrazzuolo, A., Massacci, A., Maggio, R., Confroti, A., Compagnone, M., Aurisicchio, L., Ciliberto, G., and Palombo, F. (2021). Antitumor efficacy of a neoantigen cancer vaccine delivered by electroporation is influenced by microbiota composition. Oncoimmunology *10*, 1898832. https://doi.org/10.1080/2162402X.2021.1898832.

24. Dobin, A., Davis, C.A., Schlesinger, F., Drenkow, J., Zaleski, C., Jha, S., Batut, P., Chaisson, M., and Gingeras, T.R. (2013). STAR: ultrafast universal RNA-seq aligner. Bioinformatics *29*, 15–21. https://doi.org/10.1093/bioinformatics/bts635.

25. Love, M.I., Huber, W., and Anders, S. (2014). Moderated estimation of fold change and dispersion for RNA-seq data with DESeq2. Genome Biol. *15*, 550. https://doi.org/10.1186/s13059-014-0550-8.

26. Zhang, Y., Parmigiani, G., and Johnson, W.E. (2020). ComBat-seq: batch effect adjustment for RNA-seq count data. NAR Genom. Bioinf. *2*, lqaa078. https://doi.org/10.1093/nargab/lqaa078.

27. Leek, J.T., Johnson, W.E., Parker, H.S., Jaffe, A.E., and Storey, J.D. (2012). The sva package for removing batch effects and other unwanted variation in high-throughput experiments. Bioinformatics *28*, 882–883. https://doi.org/10.1093/bioinformatics/bts034.

28. Korotkevich, G., Sukhov, V., Budin, N., Shpak, B., Artyomov, M. N., and Sergushichev, A. (2016). Fast gene set enrichment analysis. Preprint at bioRxiv. https://doi.org/10.1101/060012.

29. Chen, Z., Quan, L., Huang, A., Zhao, Q., Yuan, Y., Yuan, X., Shen, Q., Shang, J., Ben, Y., Qin, F.X.-F., and Wu, A. (2018). seq-ImmuCC: cell-centric view of tissue transcriptome measuring cellular compositions of immune microenvironment from mouse RNA-Seq data. Front. Immunol. *9*, 1286. https://doi.org/10.3389/fimmu.2018.01286.

30. Bittencourt, S.A. (2010). FastQC: A Quality Control Tool for High Throughput Sequence Data (Babraham Bioinformatics).

31. Karolchik, D., Hinrichs, A.S., Furey, T.S., Roskin, K.M., Sugnet, C.W., Haussler, D., and Kent, W.J. (2004). The UCSC Table Browser data retrieval tool. Nucleic Acids Res. *32*, D493–D496. https://doi.org/10.1093/nar/gkh103.

OMTON, Volume *34*

Supplemental information

Intramuscular plasmid DNA electroporation sequesters neoantigen-specific CD8$^+$ T cells in treated muscle and limits tumor infiltration

Bianchi Andrea, Esposito Mauro, Giacomelli Tiziano, Esposito Ilaria, Tonini Claudia, Aurisicchio Luigi, and Palombo Fabio

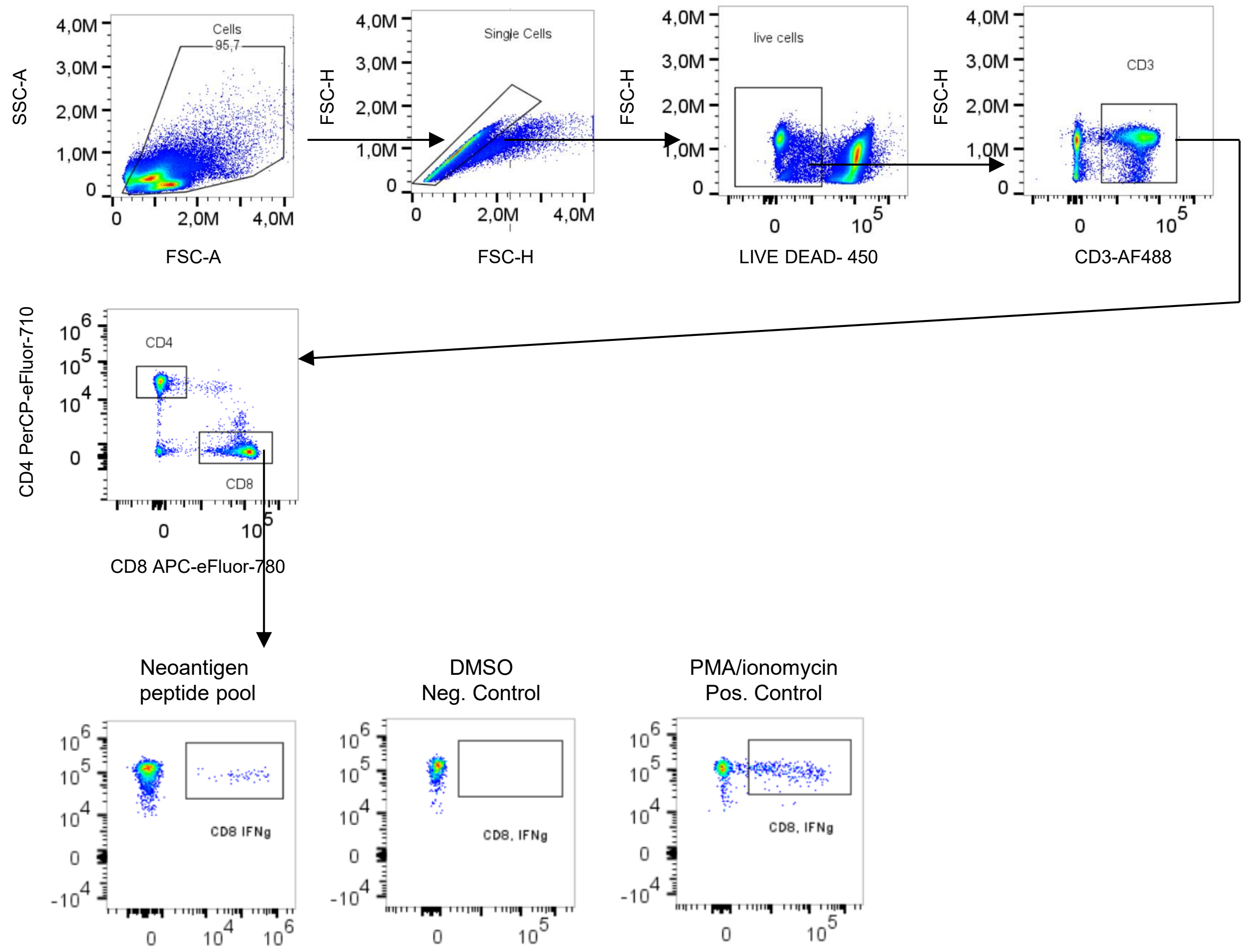

Figure S1. Representation of the gating strategy for neoantigen-specific T cells in blood sample. Isolated PBMCs were re-stimulated with the neoantigen peptide pool, DMSO (as negative control),or PMA/ionomycin (as positive control) and $CD3^{+}$ $CD8^{+}$ $IFN\gamma^{+}$ identified as consecutive gating strategy.

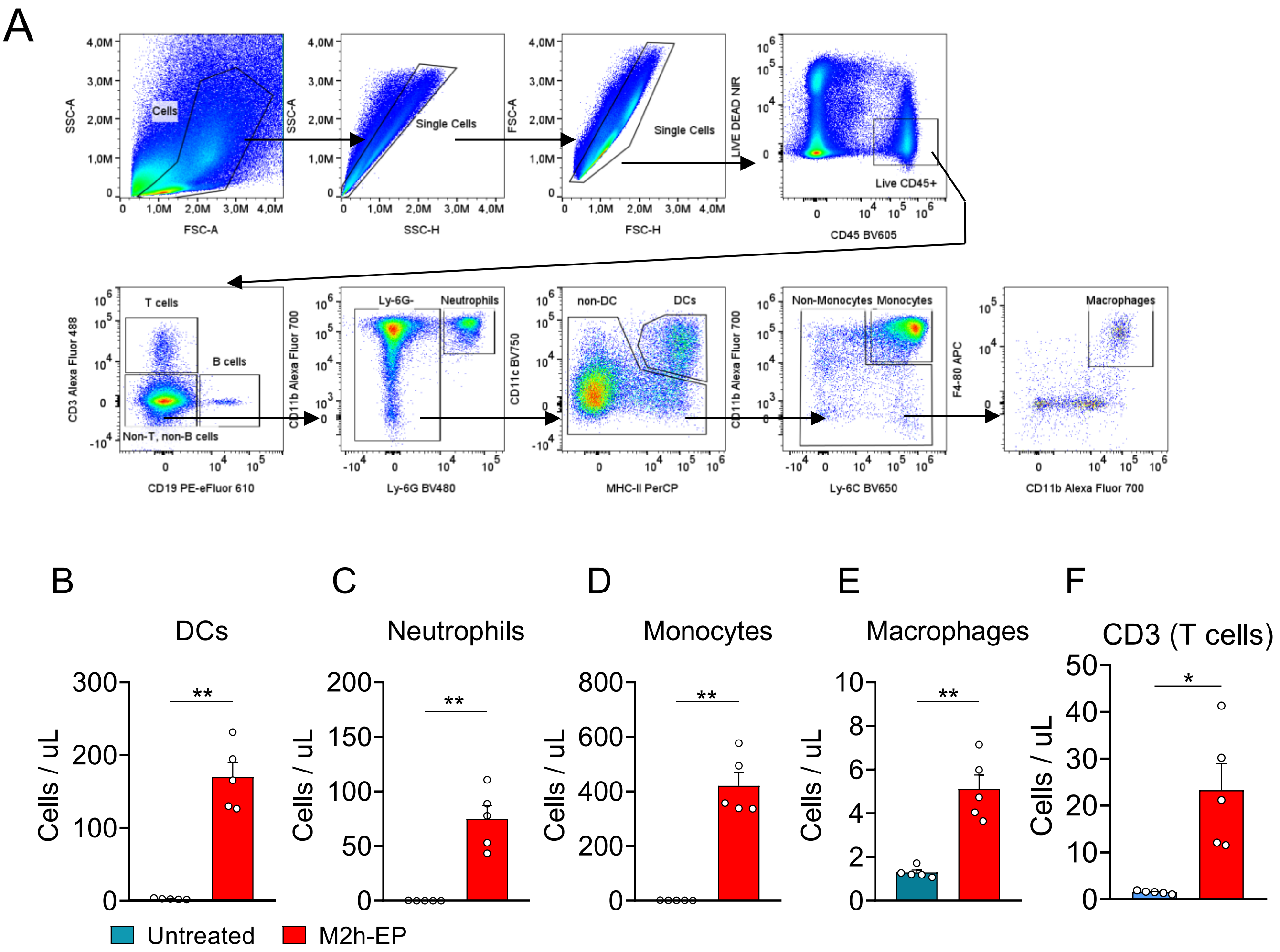

Figure S2. Representation of the gating strategy for innate and adoptive immune cells. Dendritic cells (DCs), Neutrophils, Monocytes, Macrophages, and CD3 T Cells population were anayzed in vaccinated muscles following the depicted gaing strategy 24h after vaccination (**A**) with relative quantification (**B**, **C**, **D**, **E, F**), n=5 (unpaired two-tailed t-test). Data are presented as mean ± SEM. *p < 0.05, **p < 0.01, ***p < 0.001, ****p < 0.0001; ns, non-significantly different.

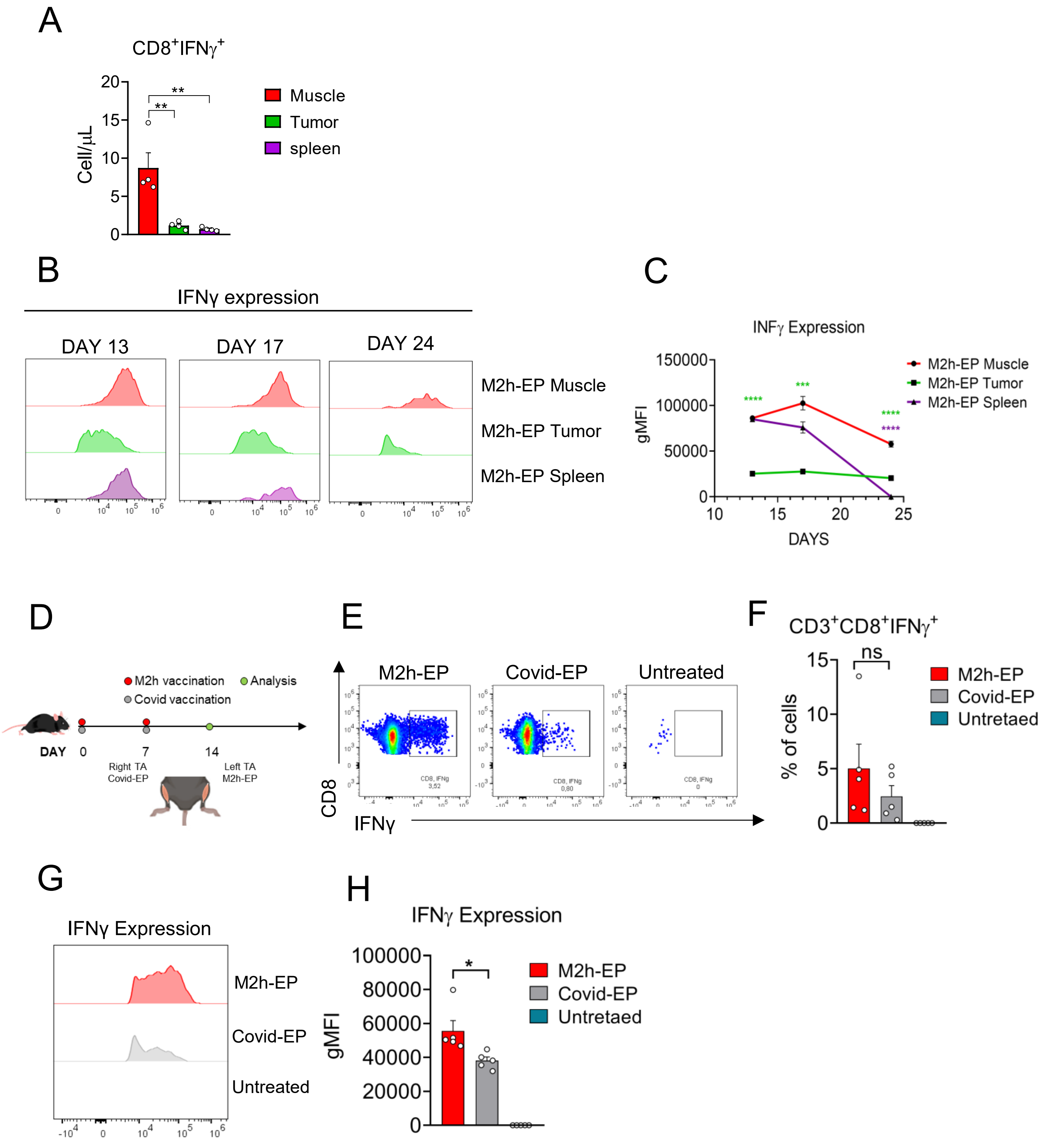

Figure S3. Quantification of neoantigen-specific T cells in different tissues. Quantification of total neoantigen-specific CD8+ T-cell (CD8+ IFNγ+) measured as cell/µL (**A**). Geometric mean fluorescence intensisty (gMFI) in CD8+ IFNγ+ T cells (**B**) and relative quantification (**C**), n= 4-5. (Ordinary one way Anova for multiple comparison). Vaccination scheme for secondary inflammation site for depot effect (**D**). Representative images of IFNγ ICS analysis (**E**) and relative quantification (**F**), n=5 (unpaired two-tailed t-test between). IFNγ expression expressed as gMFI in CD8+ CD8+ IFNγ+ T cells (**G**) and relative quantification (**H**), n= 4-5 (unpaired two-tailed t-test). All data are reported as mean ± SEM. *p < 0.05, **p < 0.01, ***p < 0.001, ****p < 0.0001 and ns= not statistically significant

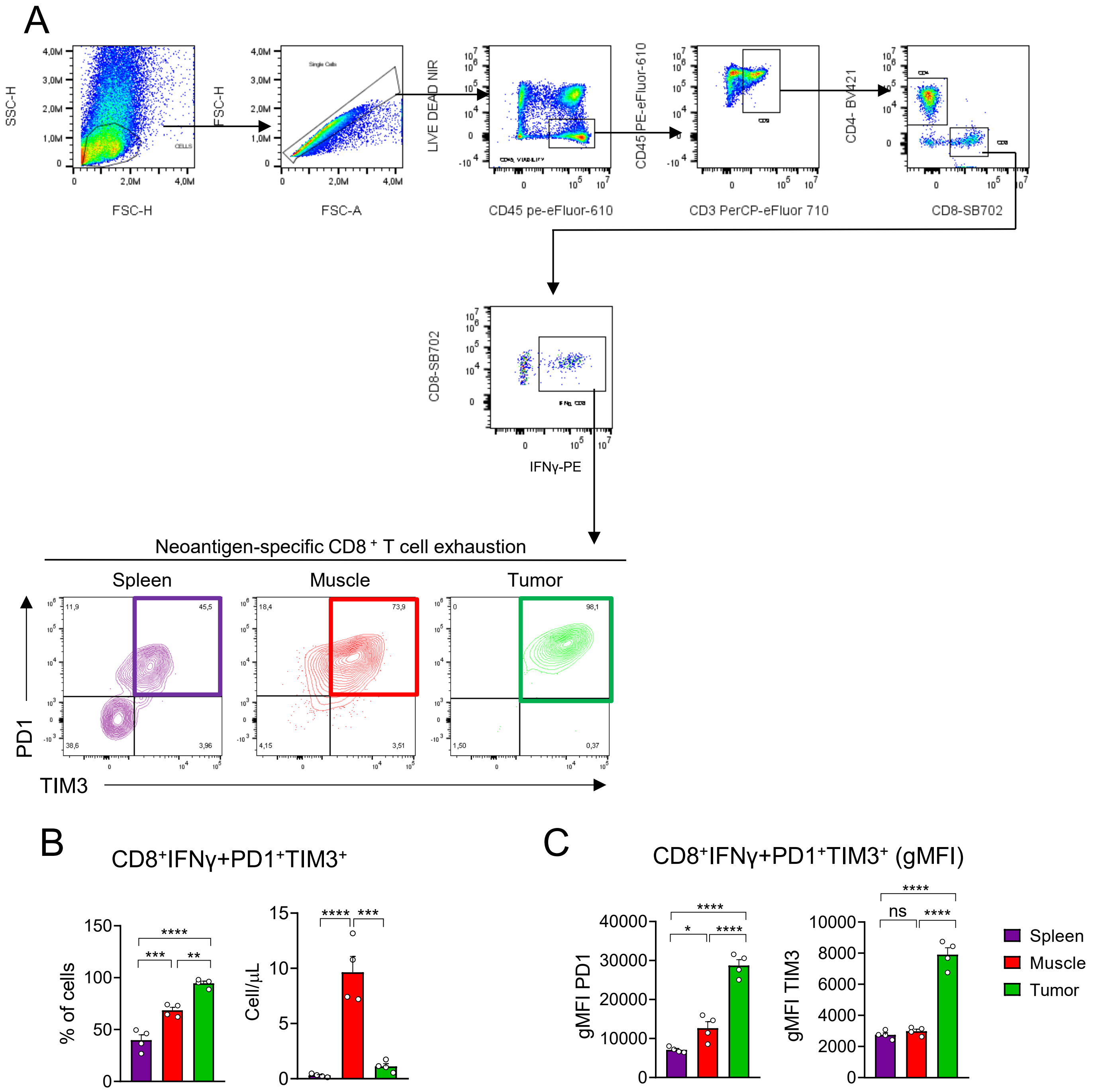

Figure S4. Flow cytometry analysis of neoantigen-specific CD8 $^+$ T cell exhaustion. Representation of the gating strategy for the PD1 and TIM3 expression in neoantigen-specific CD8$^+$IFNγ$^+$ T cells in spleen, muscle and tumor on day 13 (**A**). Percentage and total number of CD8$^+$IFNγ $^+$PD1$^+$TIM3$^+$ (**B**). gMFI of PD1 and TIM3 in CD8$^+$IFNγ+PD1$^+$TIM3$^+$ (**C**). All data (n=4) are reported as mean ± SEM. **p* < 0.05, ***p* < 0.01, ****p* < 0.001, *****p* < 0.0001 and ns= not statistically significant (Ordinary one way Anova for multiple comparison).
